# An inverse fitting strategy to determine the constrained mixture model parameters: application in patient-specific aorta

**DOI:** 10.3389/fbioe.2023.1301988

**Published:** 2023-11-20

**Authors:** Álvaro Navarrete, Andrés Utrera, Eugenio Rivera, Marcos Latorre, Diego J. Celentano, Claudio M. García-Herrera

**Affiliations:** ^1^ Departamento de Ingeniería Mecánica, Universidad de Santiago de Chile, USACH, Santiago de Chile, Chile; ^2^ Center for Research and Innovation in Bioengineering, Universitat Politècnica de València, València, Spain; ^3^ Departamento de Ingeniería Mecánica y Metalúrgica, Pontificia Universidad Católica de Chile, Santiago de Chile, Chile

**Keywords:** constrained mixture, arterial wall, deposition stretch, material parameters, finite element method

## Abstract

The Constrained Mixture Model (CMM) is a novel approach to describe arterial wall mechanics, whose formulation is based on a referential physiological state. The CMM considers the arterial wall as a mixture of load-bearing constituents, each of them with characteristic mass fraction, material properties, and deposition stretch levels from its stress-free state to the *in-vivo* configuration. Although some reports of this model successfully assess its capabilities, they barely explore experimental approaches to model patient-specific scenarios. In this sense, we propose an iterative fitting procedure of numerical-experimental nature to determine material parameters and deposition stretch values. To this end, the model has been implemented in a finite element framework, and it is calibrated using reported experimental data of descending thoracic aorta. The main results obtained from the proposed procedure consist of a set of material parameters for each constituent. Moreover, a relationship between deposition stretches and residual strain measurements (opening angle and axial stretch) has been numerically proved, establishing a strong consistency between the model and experimental data.

## 1 Introduction

Numerous investigations have reported the presence of residual stresses on arteries (Chuong and Eason, 1986), whose effect is relevant to maintaining the homeostatic state in the cardiovascular system, preventing significant intramural stress gradients on the arterial wall (Rachev and Hayashi, 1999; Wang and Gleason, 2010; Peña et al., 2015; Sigaeva et al., 2019). Its presence may be explained by non-uniform growth and remodeling processes (Fung, 1991; Bellini et al., 2014), where the stress distribution and magnitude can be altered with respect to normal levels, in face of diseases, aging or injuries (Fung, 1991; Cardamone et al., 2009; Horný et al., 2017). The residual stresses on an artery are manifested when it is extracted from its physiological state and subsequently cut, evidencing geometrical changes concerning its initial shape. Specifically, a shortening is experienced in the axial direction and, when the arterial ring-shape in an *ex-vivo* state is cut along the thickness, an opening movement can be observed. These changes from the initial configuration of the artery proves the presence of residual stresses. In practice, experimental information about residual deformation is obtained via pre-stretching and ring-opening tests (Navarrete et al., 2020; Rivera et al., 2020) where, by assuming a stress-free geometry as referential configuration, the residual stresses can be determined by an indirect way through numerical modeling of the closure ring simulation (García-Herrera et al., 2016). The artery shortening and radial cut generate a nearly complete residual strain and stress release. However, different authors questioned this statement, adding alternative measures to characterize a complete stress-free state, by either measuring a longitudinal opening (Wang and Gleason, 2010), separating arterial layers (Peña et al., 2015) or formulating novel constitutive theories that do not require an experimental stress-free state due to the physical impracticality of generating a full release of residual stresses (Ciarletta et al., 2016).

The Constrained Mixture Model (CMM) appears as a phenomenological approach for soft tissue mechanics in which different aspects related to growth and remodeling (G&R) phenomena are considered. These aspects are mainly referred to the time-dependent production of arterial constituents to different stressed configurations, whose activity is driven by a mechanobiological response of cell activity (Humphrey and Rajagopal, 2002). Unlike classical approaches, which consider stress-free as a referential configuration (Maes et al., 2019), the CMM is based on an *in-vivo* referential geometry (pressurized and axially loaded). This reference configuration avoids the questioning mentioned above about the stress-free state and the lack of information about the compressive response of each specific constituent (Bellini et al., 2014). A fundamental feature of this model is the consideration that the artery wall is composed of different load-bearing constituents: elastin, collagen, and smooth muscle cells (Valentín et al., 2013; Mousavi and Avril, 2017); each one contributing to its overall behavior, using concepts related to the theory of mixtures and homogenization (Humphrey and Rajagopal, 2002). The CMM framework allows accounting for the weighted contribution of strain energy and stress for each constituent, according to kinetic considerations related to their mass evolution (production and removal). In this way, previous studies on arteries revealed a high rate of production of elastin in the perinatal period, followed by a low turnover rate during maturity (Humphrey and Rajagopal, 2002). In contrast, collagen is continuously produced and degraded (short half-life), which may be further reduced by arterial diseases, like hypertension or aneurysms (Cyron and Humphrey, 2017). On the other hand, the CMM takes into consideration potential structural changes of each component; for example, through degradation of their material properties (Latorre and Humphrey, 2020), or alteration of its stressed configuration over time (Laubrie et al., 2022). Particular attention is put on this last point since, as the *in-vivo* configuration is taken as a referential state, the definition of a characteristic deposition stretch (pre-strain) by each constituent is necessary, which quantifies its deformation from a stress-free (natural) configuration to the referential state. The theoretical basis of the CMM explains the presence of residual stresses as a result of the difference in stress states between constituents deposited to the material and those of the material itself (Humphrey and Rajagopal, 2002).

As stated by the overview work performed by (Morin and Avril, 2015), which is focused on inverse-nature problems applied to the arterial mechanical behavior, the proper calibration of the mechanobiological model parameters is a challenge to be addressed. One of the main tasks concerning models that consider an *in-vivo* reference state is the determination of its pre-stress field via pre-strain, due to the assumption of material elasticity (Maas et al., 2016). Therefore, it is necessary to conceive methods to determine the desired parameters, with the limitation associated to the lack of information about the stress-free state. Related to this point, different authors have developed computational frameworks to solve this problem (Weisbecker et al., 2014). utilized information about the *in-vivo* reference configuration (geometry and loads) to determine pre-stretch gradients present in this configuration, based on an iterative procedure that involves solving an inverse finite element problem. The work of (Maas et al., 2016) was focused on determining pre-stresses and pre-strains of biological tissues in a finite element context by an algorithm that verifies equilibrium in the face of potential incompatibilities concerning initial pre-strain values.

Many efforts have been put into the numerical implementation of the CMM and its application in G&R. The increasing need to characterize the material parameters to achieve practical use of this model applied to specific patients has contemporary significance. One of the main limitations is the difficulty at the time of determining material parameters and deposition stretch values directly from experimental data, because of the macroscopic nature of the most commonly used testing methods. In this regard (Latorre and Humphrey, 2018), developed a progressive nonlinear regression to determine elastic and G&R parameters of a CMM using experimental data from (Wu et al., 2014; Bersi et al., 2016) for a particular mouse model of hypertension for which information was available on the time course of changes in blood pressure, wall composition, material properties, and inflammation (Mousavi and Avril, 2017). developed a finite element implementation to determine the pre-strain tensor of elastin, which is subjected to high stretches due to its initial deposition in the perinatal period in conjunction with its long half-life. This implementation was applied to obtain residual stresses and to validate the opening angle on an idealized geometry, using experimental data of a murine artery segment. In addition, the determination of the residual stress field and material parameters using the CMM was evaluated on the patient-specific model of a human ascending thoracic aorta. Within the study performed by (Laubrie et al., 2022), an algorithm based on (Maas et al., 2016) was implemented to derive the spatial distribution of pre-strain, considering the combined effect of elastin and ground substances of extracellular matrix on a patient-specific aortic arch (Maes et al., 2019). introduced an iterative fitting method, determining a set of material parameters for the CMM under the assumption of fixed deposition stretch values.

In this context, taking previous studies as a basis, we propose in the present work a numerical approach to perform an integrated characterization on these values. To achieve this objective, the CMM has been implemented in the FEBio finite element framework (Maas et al., 2012). The model characterization has been performed using experimental mechanical data reported by (Rivera et al., 2021) on a newborn lamb-specific descending thoracic aorta. The CMM is fitted to fulfill and correlate with experimental data obtained from the following tests: ring-opening, pre-stretch, pressurization-stretch and uniaxial tensile using samples in circumferential and longitudinal directions.

The contents of the manuscript are organized as follows: Section 2 presents the CMM background, details about experimental procedure and the information considered from it, along with the description of the iterative-fitting procedure proposed. Section 3 shows the main results obtained after applying this procedure on a patient-specific case, and a sensitivity analysis of the variation of relevant parameters. Section 4 includes an extensive discussion and analysis of numerical results and performance of the proposed procedure. Finally, further remarks and future research lines are identified.

## 2 Material and methods

In this section, the aspects related to the mathematical formulation of the Constrained Mixture Model (CMM) (Section 2.1) along with the procedure to perform the corresponding fitting (Section 2.3) are exhibited below. It is worth mentioning that experimental data used for this aim is totally obtained from previous studies (detailed in Section 2.2), and this work is particularly focused on the numerical analysis of this data.

### 2.1 Constrained mixture model (CMM)

**FIGURE 1:**
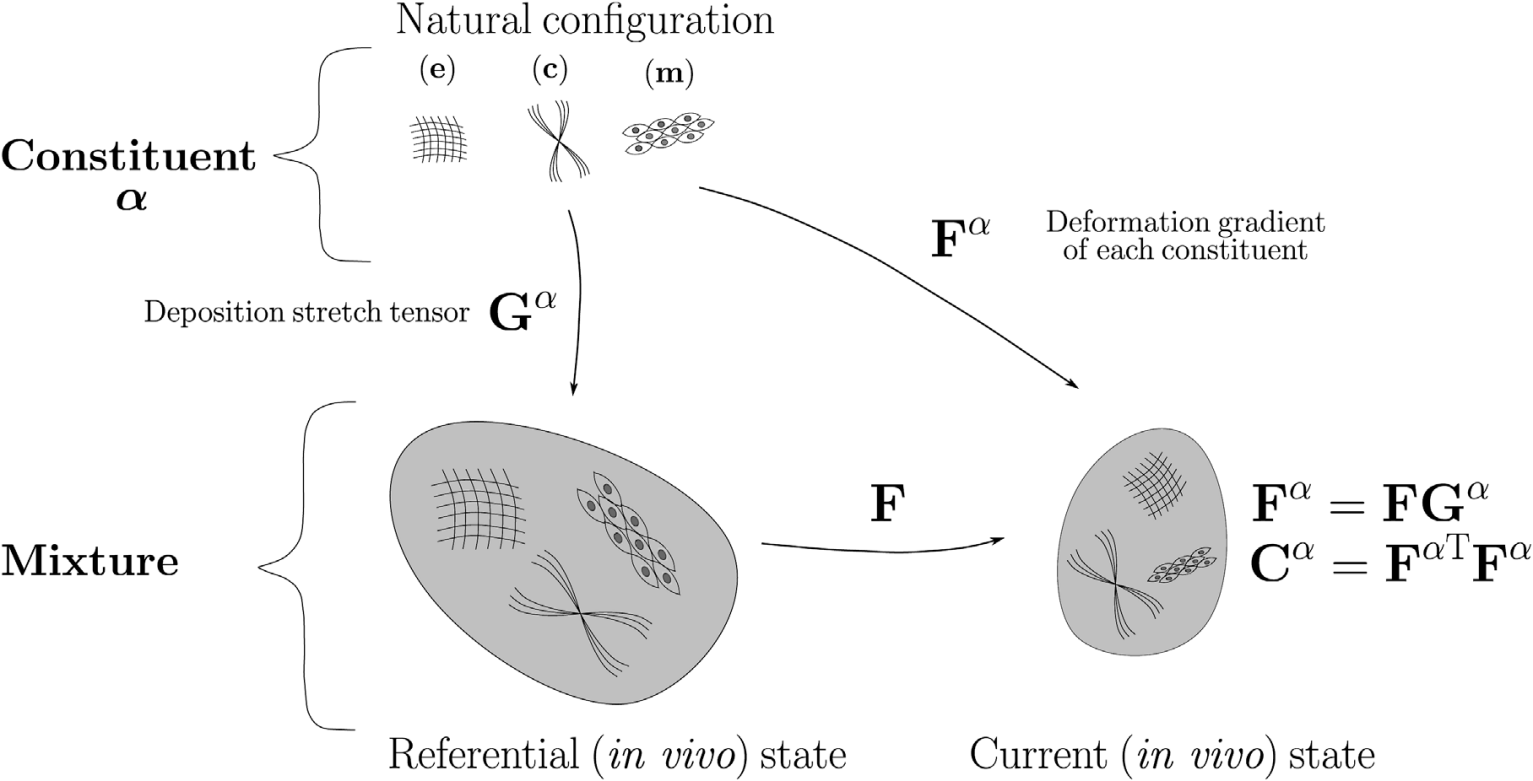
Schematic representation of CM model.

According to Figure 1, the CMM considers that the arterial wall mechanics is influenced by a mixture of the major families of load-bearing constituents (*α*): elastin (e), collagen (c) and smooth muscle (m) (Wang et al., 2016), each one associated with its corresponding mass fraction (*ϕ* ^*α*^; *α* = e, c, m). Each constituent is deposited into the mixture, from its theoretical stress-free (natural) configuration to the *in-vivo* configuration (with pressurization and axial stretch at physiological levels). The deformation gradient experienced by each family of constituents *α* is, under this condition, named as deposition stretch (G^*α*^). These constituents are constrained to deform with the mixture in the face of subsequent changes in the mechanical loads on the whole arterial wall, whose deformation gradient is denoted by F. Therefore, it is possible to define the deformation gradient of each specific constituent (F^*α*^) when the arterial wall changes its configuration with reference to homeostatic conditions as:

$$F^{\alpha }=FG^{\alpha }\;\;\alpha =e,c,m$$ (1)

The arterial wall is often considered an incompressible hyperelastic material (Chagnon et al., 2015). In this sense, the theory of hyperelasticity establishes that the stress-strain relationship is adequately modeled with a consistent strain energy function (W). To enforce incompressibility in the finite element context, the formulation of W is considered in its nearly-incompressible expression (Mousavi and Avril, 2017; Latorre and Humphrey, 2020). This condition is associated with an isochoric-volumetric decomposition of W, whose particularization to the CMM is stated by:

$$W=\underset{\alpha =e,c,m}{\sum }\phi^{\alpha }{\bar{W}}^{\alpha }\left({\bar{C}}^{\alpha }\right)+U\left(J\right)$$ (2)

where the overall isochoric contribution corresponds to the sum of each specific-constituent strain energy function (left term of Eq. (2)), which in turn is dependent on the isochoric part of the right Cauchy-Green strain tensor ${\bar{C}}^{\alpha }={\bar{F}}^{\alpha T}{\bar{F}}^{\alpha }$ and ${\bar{F}}^{\alpha }=J^{-1/3}F^{\alpha }$ , with J as the determinant of deformation gradient F $\left(J=\det\left(F\right)\right)$ . The volumetric contribution (right term of Eq. (2)) is defined by an appropriate penalty strain energy function $\left(U\left(J\right)\right)$ as:

$$U\left(J\right)=0.5\kappa \left(\frac{J^{2}-1}{2}-\ln\left(J\right)\right)$$ (3)

where *κ* is a penalty parameter related with the bulk modulus which, in a near incompressibility condition, is considered much greater than the shear modulus defined within the isochoric term of W.

Focusing on the definition of ${\bar{W}}^{\alpha }$ by each constituent, the isochoric strain energy function of the elastin (*e*) is defined as a neo-Hookean material:

$$\begin{array}{cc} {\bar{W}}^{e}\left({\bar{C}}^{e}\right) & =\frac{c^{e}}{2}\left({\bar{C}}^{e}:I-3\right)\\ & =\frac{c^{e}}{2}\left({\bar{I}}_{1}^{e}-3\right) \end{array}$$ (4)

where I is the identity tensor, c^*e*^ represents the shear modulus, and ${\bar{I}}_{1}^{e}$ is the first invariant of ${\bar{C}}^{e}$ . Here, *κ* is defined five hundred times c^*e*^ to enforce the incompressibility condition (det F = J ≈ 1) (Latorre and Humphrey, 2020).

The strain energy function of the isochoric term of the collagen (c) $\left({\bar{W}}^{\text{c}}\right)$ is defined in relation to its characteristic arrangement of fibers as:

$$\begin{array}{cc} {\bar{W}}^{c}\left({\bar{C}}^{c}\right) & =\frac{k_{1}^{c}}{2k_{2}^{c}}\exp\left[k_{2}^{c}{\left(\left({\bar{C}}^{c0}:d^{c0}⊗d^{c0}\right)-1\right)}^{2}\right]-1\\ & +\frac{k_{1}^{c}}{2k_{2}^{c}}\exp\left[k_{2}^{c}{\left(\left({\bar{C}}^{c1}:d^{c1}⊗d^{c1}\right)-1\right)}^{2}\right]-1\\ & =\frac{k_{1}^{c}}{2k_{2}^{c}}\underset{i=4,6}{\sum }\left(\exp\left[k_{2}^{c}{\left({\bar{I}}_{i}^{c}-1\right)}^{2}\right]-1\right) \end{array}$$ (5)

where $k_{1}^{c}$ and $k_{2}^{c}$ are material parameters; d^*c*0^ and d^*c*1^ define the mean orientation of two families of fibers symmetrically arranged. In cylindrical coordinates (r, *θ*, z): $d^{c0}=\left[0\;\;\;\sin\left(\beta \right)\;\;\;\cos\left(\beta \right)\right]$ and $d^{c1}=\left[0\;\;\;-\sin\left(\beta \right)\;\;\;\cos\left(\beta \right)\right]$ , with *β* being the angle measured with respect to the longitudinal direction (z) in the z*θ* plane. Finally, ${\bar{I}}_{4}^{c}$ and ${\bar{I}}_{6}^{c}$ are the pseudoinvariants of ${\bar{C}}^{c0}$ and ${\bar{C}}^{c1}$ respectively, each one of them related to one specific fiber family ( ${\bar{I}}_{4}^{c}={\bar{C}}^{c0}:d^{c0}⊗d^{c0}$ and ${\bar{I}}_{6}^{c}={\bar{C}}^{c1}:d^{c1}⊗d^{c1}$ ) (Maes et al., 2019). It is worth mentioning that the fiber definition is performed on the referential geometry established in the CMM (*in-vivo* state), see Figure 1 and that other works consider two additional fiber families in axial and circumferential directions to define a 4-fiber-family model ((Bellini et al., 2014; Latorre and Humphrey, 2018)).

The strain energy function of the isochoric component of the smooth muscle (m) $\left({\bar{W}}^{\text{m}}\right)$ models it as an anisotropic fibrous material (such as in the case of collagen) oriented exclusively in the circumferential direction. Its expression is given by:

$$\begin{array}{cc} {\bar{W}}^{m}\left({\bar{C}}^{m}\right) & =\frac{k_{1}^{m}}{2k_{2}^{m}}\exp\left[k_{2}^{m}{\left(\left({\bar{C}}^{m}:d^{m}⊗d^{m}\right)-1\right)}^{2}\right]-1\\ & =\frac{k_{1}^{m}}{2k_{2}^{m}}\exp\left[k_{2}^{m}{\left({\bar{I}}_{4}^{m}-1\right)}^{2}\right]-1 \end{array}$$ (6)

where $k_{1}^{m}$ and $k_{2}^{m}$ are material parameters; d^*m*^ corresponds to the smooth muscle orientation, where in this particular case $d^{m}=\left[0\;1\;0\right]$ in cylindrical coordinates, and ${\bar{I}}_{4}^{m}={\bar{C}}^{m}:d^{m}⊗d^{m}$ is the pseudoinvariant of ${\bar{C}}^{m}$ related to the fiber direction.

The deposition stretch tensor of each constituent (G^*α*^) is defined symmetrically and volume-preserving $\left(\det\left(G^{\alpha }\right)=1\right)$ . According to the nature of each constituent, G^*α*^ must be configured appropriately. In this context, the elastin matrix has characteristic values of deposition stretch in the circumferential $\left(G_{\theta }^{e}\right)$ and longitudinal $\left(G_{z}^{e}\right)$ directions, whereas the radial direction is stated to satisfy the isochoric condition. The G^*e*^ tensor is defined in cylindrical coordinates as:

$$G^{e}=\left[\begin{array}{ccc} \frac{1}{G_{\theta }^{e}G_{z}^{e}} & 0 & 0\\ 0 & G_{\theta }^{e} & 0\\ 0 & 0 & G_{z}^{e} \end{array}\right]$$ (7)

The deposition stretch tensor of each family of collagen fibers (G^*ci*^, *i* = 0, 1) and smooth muscle (G^*m*^) are defined in reference to their mean orientation (d^*ci*^ *i* = 0, 1 and d^*m*^, respectively) in cylindrical coordinates, according to (Maes et al., 2019):

$$G^{ci}=G^{c}\left(d^{ci}⊗d^{ci}\right)-\frac{1}{\sqrt{G^{c}}}\left(I-d^{ci}⊗d^{ci}\right)\;i=0,1$$ (8)

$$G^{m}=G^{m}\left(d^{m}⊗d^{m}\right)-\frac{1}{\sqrt{G^{m}}}\left(I-d^{m}⊗d^{m}\right)$$ (9)

where G^*c*^ and G^*m*^ correspond to the deposition stretch values along the respective fiber directions.

The definition of the different deformation gradients related to the CMM allows us to define the Second Piola-Kirchhoff stress tensor (S) as:

$$\begin{array}{cc} S=2\frac{\partial W}{\partial C}= & 2\phi^{e}\frac{\partial {\bar{W}}^{e}}{\partial {\bar{I}}_{1}^{e}}\frac{\partial {\bar{I}}_{1}^{e}}{\partial C}+2\phi^{c}\left[\frac{\partial {\bar{W}}^{c}}{\partial {\bar{I}}_{1}^{c}}\frac{\partial {\bar{I}}_{1}^{c}}{\partial C}+\frac{\partial {\bar{W}}^{c}}{\partial {\bar{I}}_{4}^{c}}\frac{\partial {\bar{I}}_{4}^{c}}{\partial C}+\frac{\partial {\bar{W}}^{c}}{\partial {\bar{I}}_{6}^{c}}\frac{\partial {\bar{I}}_{6}^{c}}{\partial C}\right]\\ & +2\phi^{m}\left[\frac{\partial {\bar{W}}^{m}}{\partial {\bar{I}}_{4}^{m}}\frac{\partial {\bar{I}}_{4}^{m}}{\partial C}\right]+2\frac{\partial U\left(J\right)}{\partial J}\frac{\partial J}{\partial C} \end{array}$$ (10)

Finally, from the expression 10, the Cauchy-stress tensor can be written as:

$$\sigma =\frac{1}{J}FSF^{T}$$ (11)

### 2.2 Experimental procedure

**FIGURE 2:**
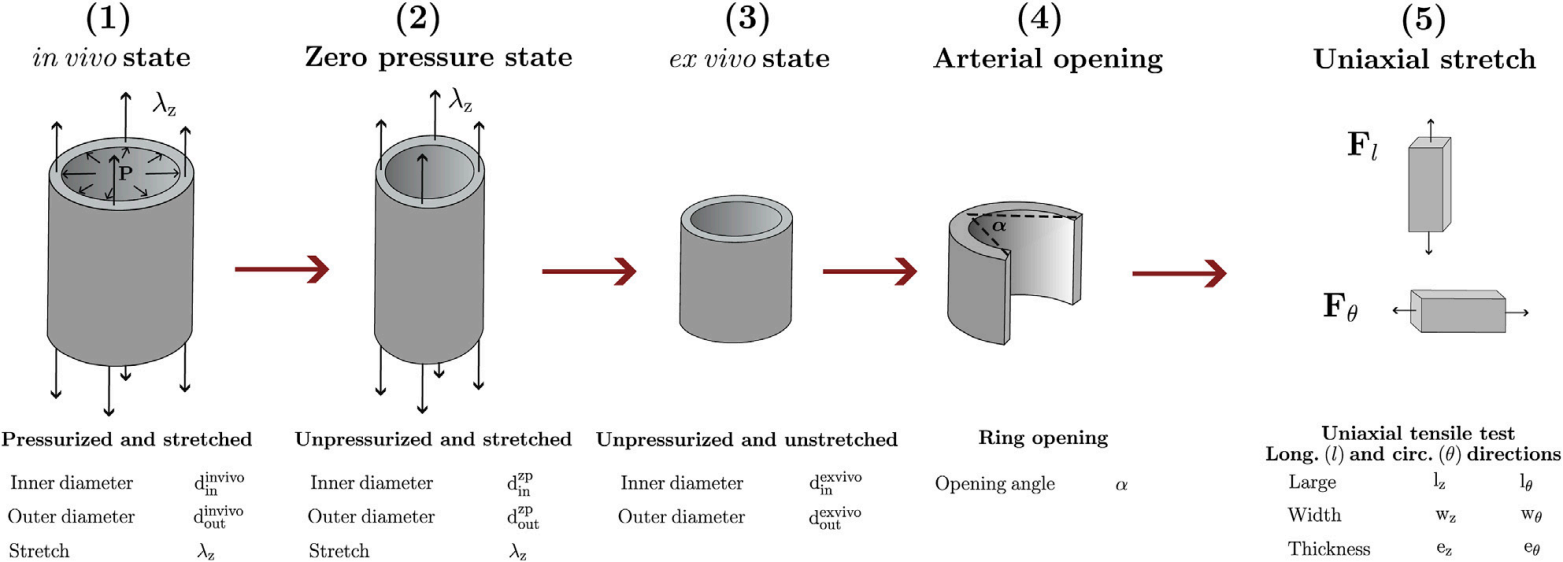
Mechanical loads and deformations to which the artery is subjected under different states: (1) in-vivo; (2) zero-pressure; (3) ex-vivo; (4) arterial opening; (5) uniaxial stretch.

The procedure described below considers the experimental data of a descending thoracic aorta of a 30-day-old newborn lamb (*Ovis Aries*) available in the works of (Rivera et al., 2020; Rivera et al., 2021). Further information about protocols and methods of measurement are detailed in both references. Figure 2 shows a scheme with the different mechanical loads and deformations to which an arterial specimen is subjected from its *in-vivo* state, to uniaxial stretch in longitudinal and circumferential directions. In this sense, the experimental procedure accounts for information of these states, via appropriate mechanical and residual deformation tests. The physiological arterial stretch *λ*_*z*_ related to *in-vivo* and zero-pressure states (steps (1) and (2), respectively) was obtained via the pre-stretching test, measuring the longitudinal shortening between the zero-pressure and *ex-vivo* states (steps (2) and (3)) (Navarrete et al., 2020). The outer diameters $d_{\text{out}}^{\text{invivo}}$ and $d_{\text{out}}^{\text{zp}}$ were registered from *in-vitro* inflation-extension tests (Rivera et al., 2021), where $d_{\text{out}}^{\text{invivo}}$ was considered under the reference pressure value, which in this case was set as the diastolic pressure (*p* = 10 kPa (Maes et al., 2019)), and $d_{\text{out}}^{\text{zp}}$ was measured at zero pressure. The experimental data of the inner $\left(d_{\text{in}}^{\text{exvivo}}\right)$ and outer $\left(d_{\text{out}}^{\text{exvivo}}\right)$ diameters in *ex-vivo* state (step (3)) was obtained from images of ring shape specimens. The inner diameter of the geometrical configuration of steps (1) $\left(d_{\text{in}}^{\text{invivo}}\right)$ and (2) $\left(d_{\text{in}}^{\text{zp}}\right)$ were obtained through the incompressibility condition (Carew et al., 1968). Step (4) represents the ring opening test (Cañas et al., 2018), from which a measure of the residual circumferential deformation described by an opening angle *α* can be calculated. Finally, from step (4), two rectangular specimens were extracted in order to perform the aforementioned uniaxial tensile test (step (5)) and obtain different sample dimensions (l_z_, w_z_, e_z_, l_*θ*_, w_*θ*_, e_*θ*_). It is worth mentioning that the mass fraction of the arterial wall constituents have been considered as fixed parameters based on referential values in the literature, specifically according to the animal model and age. Within this context, the study performed by (Wells et al., 1999) measures relative aortic elastin (*ϕ* ^e^) and collagen (*ϕ* ^c^) mass contents of 21-day-old lambs; meanwhile smooth muscle (*ϕ* ^m^) has been considered as the remaining mass fraction. All numerical values of the parameters defined above are indicated in the supplementary material file.

### 2.3 Iterative numerical-experimental fitting

Taking as basis the iterative procedure followed by (Maes et al., 2019), the material parameters $\left(mp=\left[c^{\text{e}},k_{1}^{\text{c}},k_{2}^{\text{c}},\beta ,k_{1}^{\text{m}},k_{2}^{\text{m}}\right]\right)$ and deposition stretch $\left(ds=\left[G_{\theta }^{e}\;\;\;G_{\text{z}}^{e}\;\;\;G^{\text{c}}\;\;\;G^{\text{m}}\right]\right)$ values are characterized. Both the parameters attached to mp and ds are defined in Section 2.1. This procedure takes experimental information detailed in Section 2.2, which is used as both input and validation for the numerical part of the iterative procedure.

**FIGURE 3:**
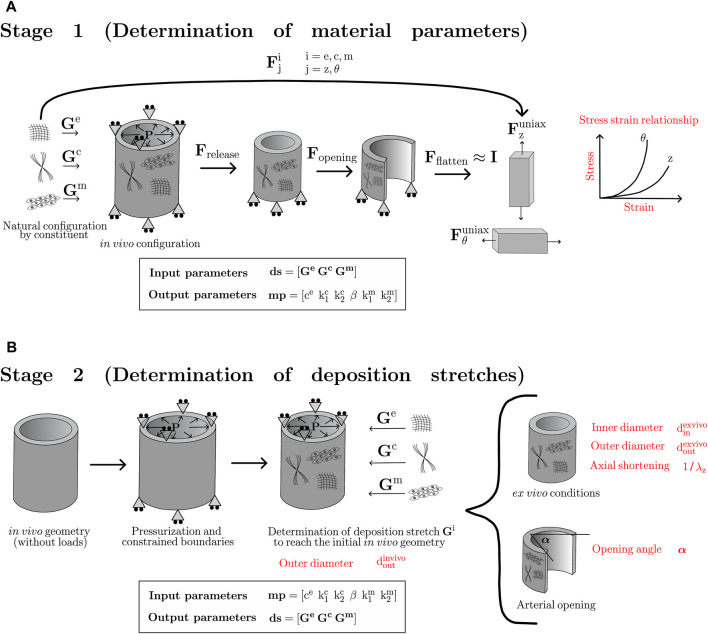
Numerical stages followed to determine: **(A)** material parameters; **(B)** deposition stretch values.

To provide a clear description of the iterative procedure, we first distinguish two main stages (Figure 3), each of them aiming to the determination of: the set of materials parameters mp (Stage 1, Figure 3A) and the deposition stretch values ds (Stage 2, Figure 3B).

#### 2.3.1 Stage 1

In Stage 1, the individual constituents are deposited from its natural configuration to the artery in its *in-vivo* state through a given set of ds (input parameters). Since the uniaxial test configuration is a flat strip of tissue, a series of deformation gradients, that allow to account for the changes occurred between the *in-vivo* and uniaxial flatten-strips, are applied to the uniaxial problem. Those gradients account for the loads release (F_release_), radial opening (F_opening_) and strip flattening (F_flatten_), where this last step is considered to produce near-zero strains on the sample strip (F_flatten_ ≈ I), due to its negligible value with respect to the deformation experienced in the previously mentioned steps (Maes et al., 2019). During the first iteration, both deformation gradients F_release_ and F_opening_ are considered to be equal to an identity tensor (I), as well as the deposition stretch parameters ds. However, in the consecutive iterations, those tensors and parameters are updated from the second stage of the proposed algorithm. The mentioned tensors, defined in cylindrical coordinates (r, *θ*, z), are given by the following expressions:

$$F_{\text{release}}=\left[\begin{array}{ccc} \lambda_{\text{release}}^{\text{r}} & 0 & 0\\ 0 & \lambda_{\text{release}}^{\theta } & 0\\ 0 & 0 & \lambda_{\text{release}}^{\text{z}} \end{array}\right]\;\;F_{\text{opening}}=\left[\begin{array}{ccc} \lambda_{\text{opening}}^{\text{r}} & 0 & 0\\ 0 & \lambda_{\text{opening}}^{\theta } & 0\\ 0 & 0 & \lambda_{\text{opening}}^{\text{z}} \end{array}\right]$$ (12)

Finally, the deformation gradient of each constituent from its natural configuration to the stretching applied in the uniaxial tensile test $\left(F_{\text{j}}^{\mathrm{uniax}}\;j=z,\theta \right)$ is stated by the following expression, according to the nomenclature of Figure 3A:

$$F_{j}^{i}=F_{j}^{\text{uniax}}F_{\text{flatten}}F_{\text{opening}}F_{\text{release}}G^{\text{i}}\;i=e,c,m\;j=z,\theta$$ (13)

Through this procedure, a set of final material parameters mp can be computed (output parameters). To this end, the experimental longitudinal and circumferential stress-strain relationships are taken as target curves, where the optimal parameters mp are determined by solving the inverse finite element problem associated with the numerical uniaxial tensile test in both directions.

#### 2.3.2 Stage 2

As shown in Figure 3B, Stage 2 considers fixed material parameters (mp) obtained as an output from Stage 1, and the *in-vivo* arterial geometry as the referential configuration, according to the fact that the constituents are deposited from its stress-free state to the mentioned state. Thus, the procedure of Stage 2 considers the reference geometry in the diastolic pressure stage, experimentally obtained by the inflation-extension test. Then, the artery is pressurized and axially constrained to reproduce *in-vivo* loads. However, geometrical dimensions reached in this step are not compatible with any real configuration. Then, the next step consists of applying the corresponding set of deposition stretches (ds), such that these values enable us to obtain the initial dimensions at which the analysis of Stage 2 began, ideally producing a near-zero dimensional change in the reference configuration. Therefore, at the end of this step, the artery has reached the referential geometry with its corresponding *in-vivo* stress field. Stage 2 ends with the release of the *in-vivo* loads, assumed to be a numerical *ex-vivo* configuration. The determination of parameters ds is performed via a finite element inverse approach, in which $d_{\text{out}}^{\text{invivo}}$ , $d_{\text{in}}^{\text{exvivo}}$ , $d_{\text{out}}^{\text{exvivo}}$ and *λ*_z_ are the target parameters, previously reported experimental data (Figure 2). Once the parameters ds have been established, they are used to perform the simulation of arterial ring-opening. At the end of the fitting procedure, F_release_ and F_opening_ are calculated, and utilized for the Stage 1 in the next iteration.

#### 2.3.3 Integrated procedure

**FIGURE 4:**
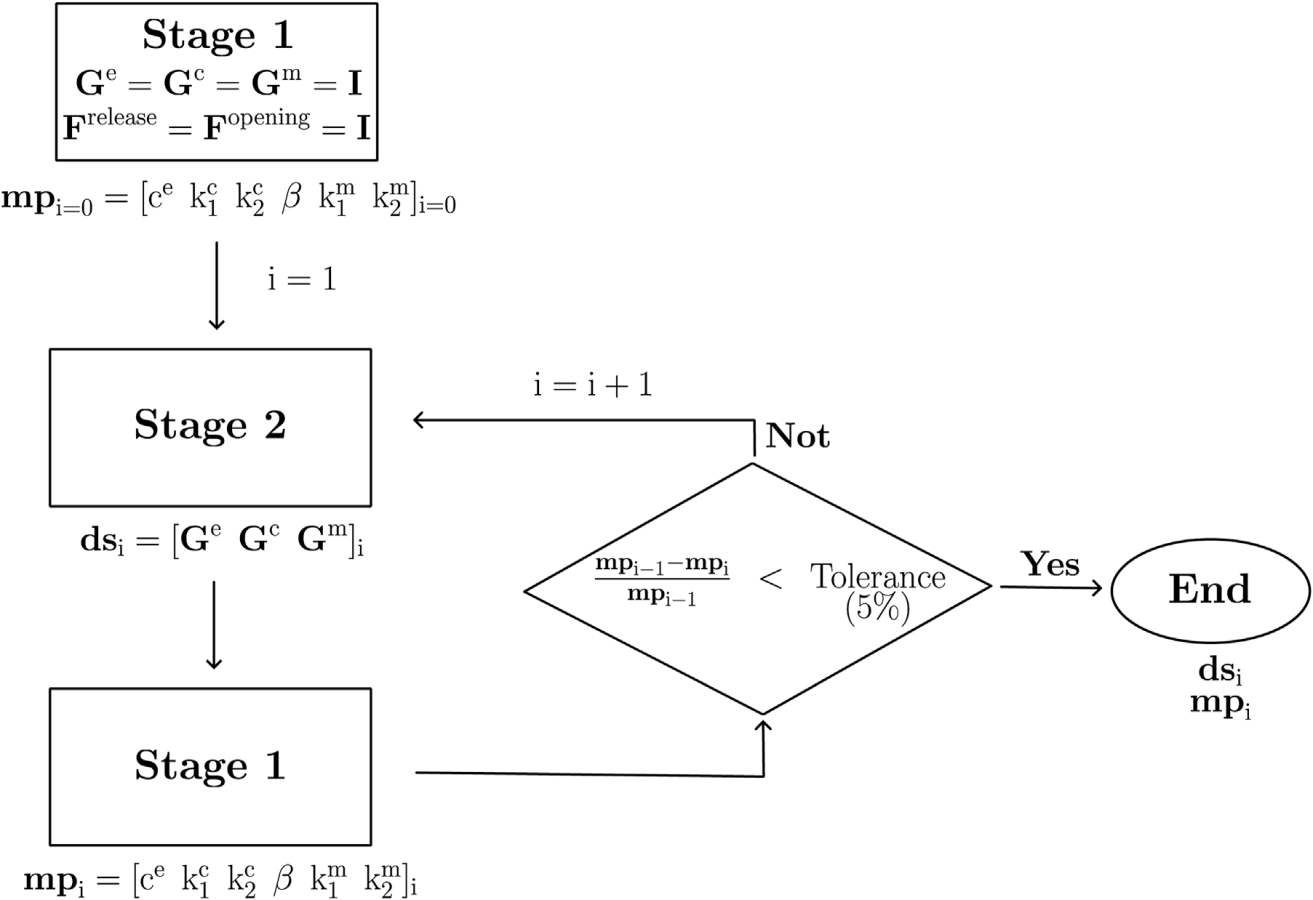
Algorithm proposed to determine the set of material parameters (mp) and deposition stretches (ds).

The two stages described above (Section 2.3.1 and Section 2.3.2) are part of the iterative procedure proposed in this work, whose goal consists of simultaneously determining material parameters (mp) and deposition stretch values (ds). As Figure 4 states, consecutive iterations from both stages are computed in a custom inverse implementation of Python’s LMFit library (Newville and Stensitzki, 2018) using FEBio results together with the standard Levenberg-Marquardt algorithm of this implementation. An experimental-numerical residual minimization approach was used to characterize both material parameters and the deposition stretches. A stop criterion on the material parameters is set to a 5% variation between the optimization iterations. The objective function f(x) used to optimize the material parameters in Stage 1 is defined as follows:

$$\underset{x\in mp}{minimize}\;f\left(x\right)=J\left(\sigma_{\text{circ}},{\hat{\sigma }}_{\text{circ}}\right)+J\left(\sigma_{\text{long}},{\hat{\sigma }}_{\text{long}}\right)$$ (14)

$$J\left(y,\hat{y}\right)=\frac{1}{n}\overset{\text{n}}{\underset{\text{i}=1}{\sum }}\frac{{\left(y-\hat{y}\right)}^{2}}{max\left(y\right)}$$ (15),

where J is the standardization function for the stress-strain residual, n represents the number of discrete points in each curve, y is the experimental value, and $\hat{y}$ the model-predicted value. In the objective function f(x), *σ*_i_ and ${\hat{\sigma }}_{\text{i}}$ are the experimental and model-predicted uniaxial stresses, respectively, where the subscript i represents the direction of the test (circumferential or longitudinal).

By another side, the objectve function g(x) utilized to optimize the desposition stretch values in Stage 2 is defined as:

$$\underset{x\in ds}{minimize}\;g\left(x\right)=K\left(d_{\text{out}}^{\text{invivo}},{\hat{d}}_{\text{out}}^{\text{invivo}}\right)+K\left(d_{\text{int}}^{\text{invivo}},{\hat{d}}_{\text{int}}^{\text{invivo}}\right)+K\left(d_{\text{out}}^{\text{exvivo}},{\hat{d}}_{\text{out}}^{\text{exvivo}}\right)+K\left(\lambda_{\text{z}},\hat{\lambda_{\text{z}}}\right)$$ (16)

$$K\left(y,\hat{y}\right)={\left(y-\hat{y}\right)}^{2}$$ (17),

where the K function defines the quadratic error used to calculate the *in vivo* and *ex-vivo* geometry error between experimental and numerical values.

The ring-shaped finite element mesh used in the numerical simulations is composed of 8-node hexahedra, with a total of 900 elements and 1952 nodes (15 radial elements, 60 circumferential elements, and one element in the axial direction). The formulation considers a three-field implementation in order to avoid numerical locking of nearly-incompressible materials (Maas et al., 2012). Moreover, the numerical simulation of the uniaxial tensile test is performed, due to the development of homogeneous stress and strain fields, by considering a single 8-noded hexahedral element.

## 3 Results

### 3.1 Patient-specific study on newborn lamb aorta

Taking the experimental results of the *in-vivo* geometry, whose values are detailed in Section 2.2, the descending thoracic aorta is modeled considering a straight cylindrical shape (Karšaj et al., 2010; Valentín et al., 2011). From the output parameters of the fitting procedure (detailed in Section 2.3), a set of material parameters mp and deposition stretch values ds were obtained. Table 1 shows the parameters used as input and those obtained from the numerical procedure (output values).

**TABLE 1:** CM model parameters, specifying those determined experimentally, by reference and as output from iterative procedure.

Parameter	Symbol	Value	Nature	References
*In-vivo* geometry and loads				
Outer diameter	$d_{\text{out}}^{\text{invivo}}$	10.31 mm	Experimental	
Inner diameter	$d_{\text{in}}^{\text{invivo}}$	8.16 mm	Experimental	
Length	l	0.2 mm		
Diastolic pressure	*p*	10 kPa	Referential	Maes et al. (2019)
Mass fractions				
Elastin	*ϕ* ^e^	0.5	Referential	Wells et al. (1999)
Collagen	*ϕ* ^c^	0.2	Referential	Wells et al. (1999)
Smooth muscle	*ϕ* ^m^	0.3	Referential	Wells et al. (1999)
Mechanical properties (mp)				
Elastin	c^e^	10.2 kPa	Output	
Collagen	$k_{1}^{\text{c}}$	52.9 kPa	Output	
	$k_{2}^{\text{c}}$	0.39	Output	
	*β*	39.7°	Output	
Smooth muscle	$k_{1}^{\text{m}}$	10.3 kPa	Output	
	$k_{2}^{\text{m}}$	0.024	Output	
Deposition stretch (ds)				
Elastin	$G_{\theta }^{\text{e}}$	1.05	Output	
	$G_{\text{z}}^{\text{e}}$	1.31	Output	
Collagen	G^c^	1.10	Output	
Smooth muscle	G^m^	1.45	Output	

**FIGURE 5:**
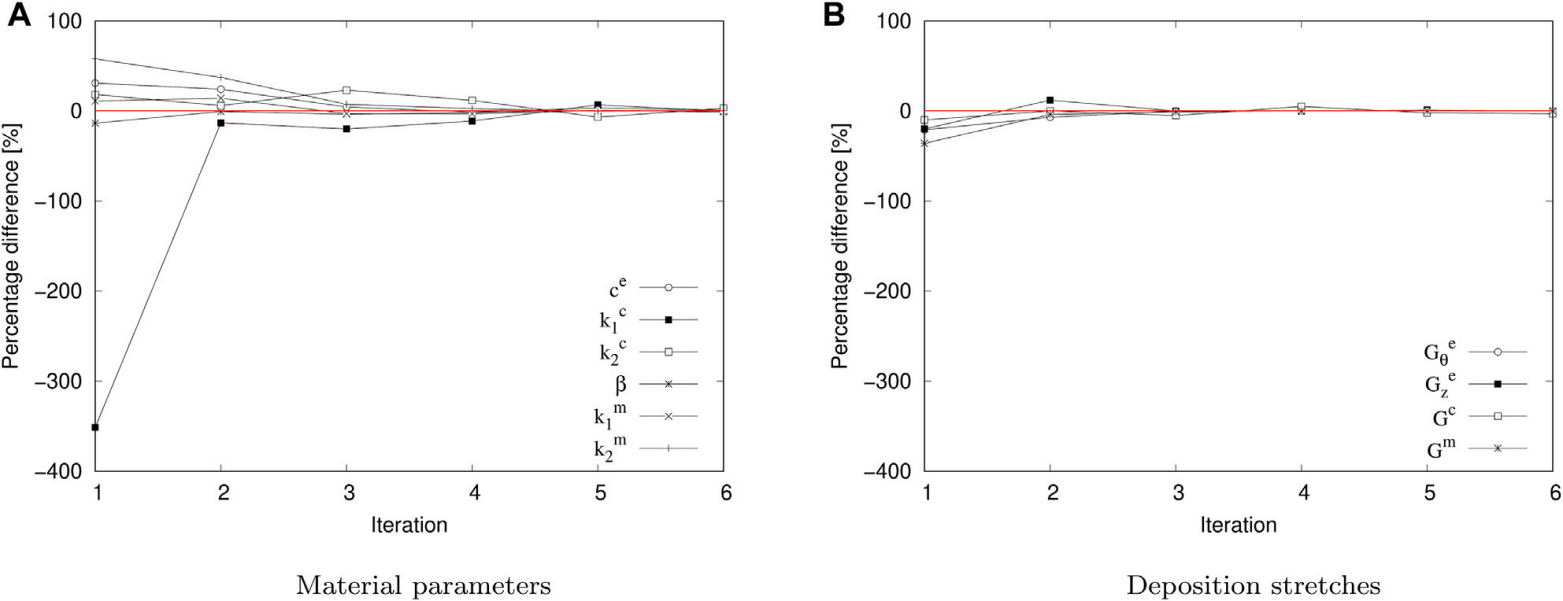
Percentage difference regarding to the successive iteration steps in the iterative fitting procedure. **(A)** material parameter relative variation (mp). **(B)** deposition stretch relative variation (ds).

Focusing on the characterization procedure, Figure 5 denotes the percentage difference of each parameter, concerning the values of the previous iteration. Figure 5A, refers to the material parameters mp that quickly approach to the 0% variation level (red line). On this basis, a remarkable case is visualized for $k_{1}^{\text{c}}$ . The percentage difference regarding the first iteration corresponds to 351%, experiencing an enormous drop in the next iteration (13%) and finishing in an absolute difference of 0.3% to the sixth iteration. In general, a similar trend can be observed in the remaining material parameter values, where at the end of the last iteration performed the highest percentage difference is close to 3% $\left(k_{2}^{\text{c}}\right)$ . Similarly, variations of deposition stretch values ds are shown for each iteration (Figure 5B). As in the previous case, a fast convergence towards the ideal target value (red line) can be observed. According to the iterative procedure (Figure 4), the percentage difference of these values is not directly considered. However, the convergence to ds denotes consistency concerning the set of determined parameters. Specifically, the maximum absolute percentage difference at the first iteration is 36% for the G^m^ parameter, while in the sixth iteration, $G_{\text{z}}^{\text{e}}$ reaches a 0.08% variation.

As stated in the description of Stage 1 (Section 2.3.1), a release and opening deformation gradient is obtained and utilized for the uniaxial extension. After the sixth iteration, the average values (average ±SEM)) across the thickness of the tissue are: $\lambda_{\text{release}}^{\text{r}}=1.51\pm 0.02$ , $\lambda_{\text{release}}^{\theta }=0.76\pm 0.02$ , $\lambda_{\text{release}}^{\text{z}}=0.88\pm 0.01$ , $\lambda_{\text{opening}}^{\text{r}}=1.04\pm 0.01$ , $\lambda_{\text{opening}}^{\theta }=1.00\pm 0.03$ and $\lambda_{\text{opening}}^{\text{z}}=0.98\pm 0.01$ . Due to the small variation of average values along the thickness (see SEM values), the consideration of a constant value along the thickness is justified.

**FIGURE 6:**
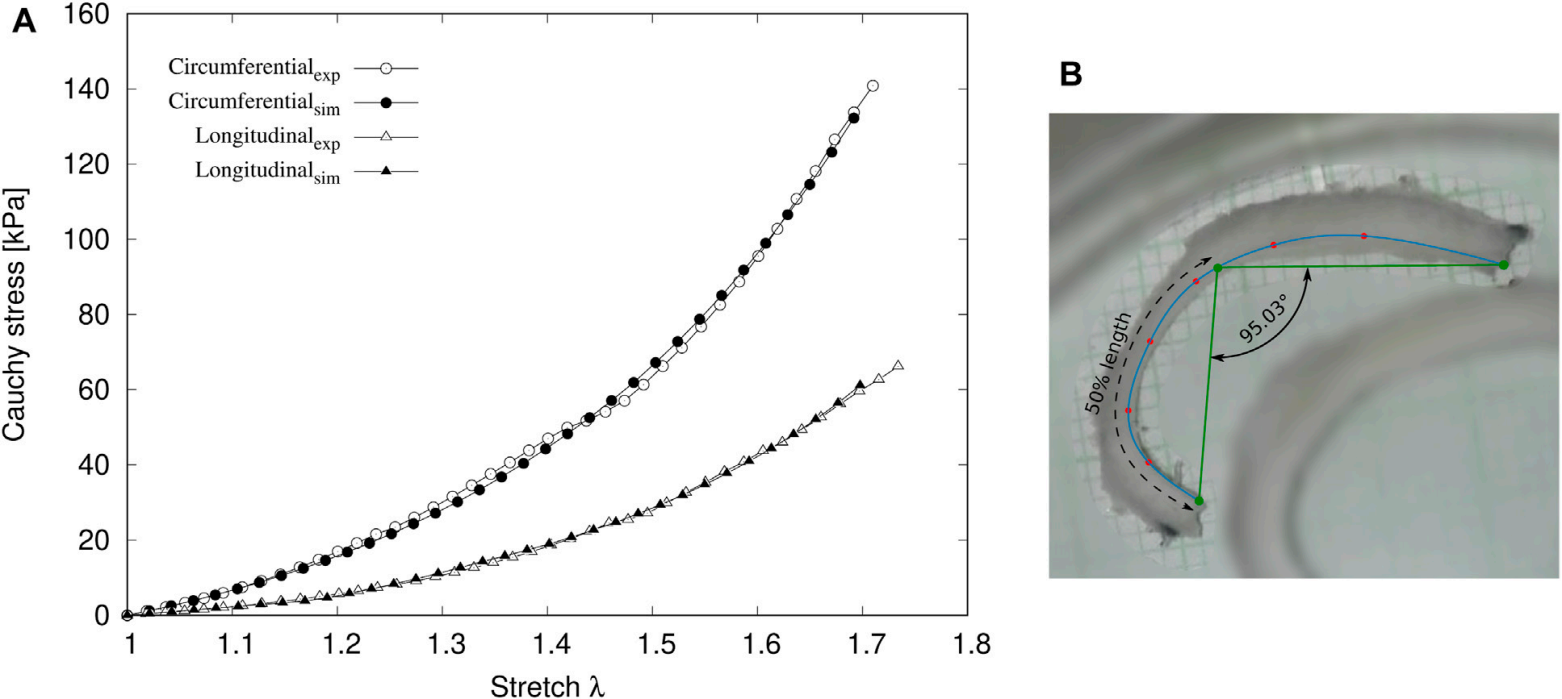
**(A)** Experimental and numerical Cauchy stress-stretch curves in circumferential and longitudinal directions. **(B)** experimental opening angle *α*.

Concerning to the target information used in Stages 1 and 2 of the iterative procedure, Figure 6A shows experimental and numerical Cauchy stress-stretch curves in circumferential and longitudinal directions, which are used in Stage 1 (Figure 3A). The numerical results are obtained from the set of material parameters (mp) after the last iteration performed. A good fit between both curves is reflected by the R-squared values: $R_{\text{circ}}^{2}=0.9979$ and $R_{\text{long}}^{2}=0.9989$ . On the other hand, the geometrical dimensions considered as target values in Stage 2 (Figure 3B), namely, $d_{\text{out}}^{\text{invivo}}$ , $d_{\text{out}}^{\text{exvivo}}$ , $d_{\text{in}}^{\text{exvivo}}$ and *λ*_z_, have been redefined for a better visualization of the ds parameters’ optimization. In this sense, the ratios between *in-vivo* and *ex-vivo* measurements: outer diameter $\left(d_{\text{out}}^{\text{invivo}}/d_{\text{out}}^{\text{exvivo}}\right)$ and thickness (e^invivo^/e^exvivo^), along with longitudinal stretch (*λ*_z_), are shown in Table 2.

**TABLE 2:** Experimental and numerical ratios between *in-vivo* and *ex-vivo* dimensions, along with its corresponding percentage difference.

Definition	Formula	Exp	Num	Diff (%)
Outer diameter variation	$d_{\text{out}}^{\text{invivo}}/d_{\text{out}}^{\text{exvivo}}$	1.10	1.19	8.24
Thickness variation (e^invivo^/e^exvivo^)	$\left(d_{\text{out}}^{\text{invivo}}-d_{\text{in}}^{\text{invivo}}\right)/\left(d_{\text{out}}^{\text{exvivo}}-d_{\text{in}}^{\text{exvivo}}\right)$	0.73	0.66	9.29
Longitudinal stretch	*λ*_z_	1.16	1.15	1.18

It is important to emphasize that the results shown in Table 2 are obtained from the last iteration performed. The main results denote that the difference between the numerical and experimental results, both for the variation of the outer diameter, thickness, and length of the artery, show differences that do not exceed 10%. Unreported results of this work indicate that once the pressurization and deposition stretches are applied on the arterial wall (see Figure 3), the geometrical variation respecting the initial *in-vivo* geometry is practically negligible (radial node displacement is in the order of 10^–2^ mm), meaning that the equilibrium between the *in-vivo* loads and the deposition process is successfully achieved.

The opening angle *α* is considered as an external parameter to check the consistency of the obtained values in the procedure followed in this work. Figure 6B exhibits the measurement of the experimental opening angle of 95.03°. According to the set of mp and ds values obtained via the iterative fitting procedure, the numerical ring opening simulation (Figure 3B) results in an opening angle of 91.39°, corresponding to an error of 3.83%.

### 3.2 Sensitivity analysis

Once the consistency of the experimental-numerical procedure has been proved (Section 3.1), a sensitivity study is performed to explore the effect of the deposition stretch and mass fraction variations in the resulting residual strains. For this analysis, the numerical results extracted from the longitudinal arterial shortening and opening angle have been considered. The variation range of the considered parameters, along with their respective referential values, are shown in Table 3. These baseline values and the material parameters mp have been taken from the final iteration of the patient-specific study of the lamb aorta, according to the values shown in Table 1. It needs to be noted that separated studies about the impact of mass fractions and deposition stretches are performed, meaning that the mass fraction sensitivity is done under a constant deposition stretch parameter set (from the last characterization iteration), and *vice versa* for the deposition stretch study (under constant mass fraction distribution, from the fixed reference values). It is essential to highlight that the mass fraction of smooth muscle *ϕ*_m_ is dependent on the remaining mass fractions, and G^c^ has been maintained in its baseline value (1.10), due to its negligible variation during the iterative procedure (Figure 5).

**TABLE 3:** Range of each parameter considered in sensitivity analysis and their respective referential values.

Parameter	Referential value	Minimum value	Maximum value
Mass fraction			
*ϕ*_e_	0.5	0.2	0.6
*ϕ*_c_	0.2	0.1	0.3
Deposition stretch			
$G_{\theta }^{\text{e}}$	1.05	1.00	1.20
$G_{\text{z}}^{\text{e}}$	1.31	1.05	1.50
G^m^	1.45	1.15	1.68

**FIGURE 7:**
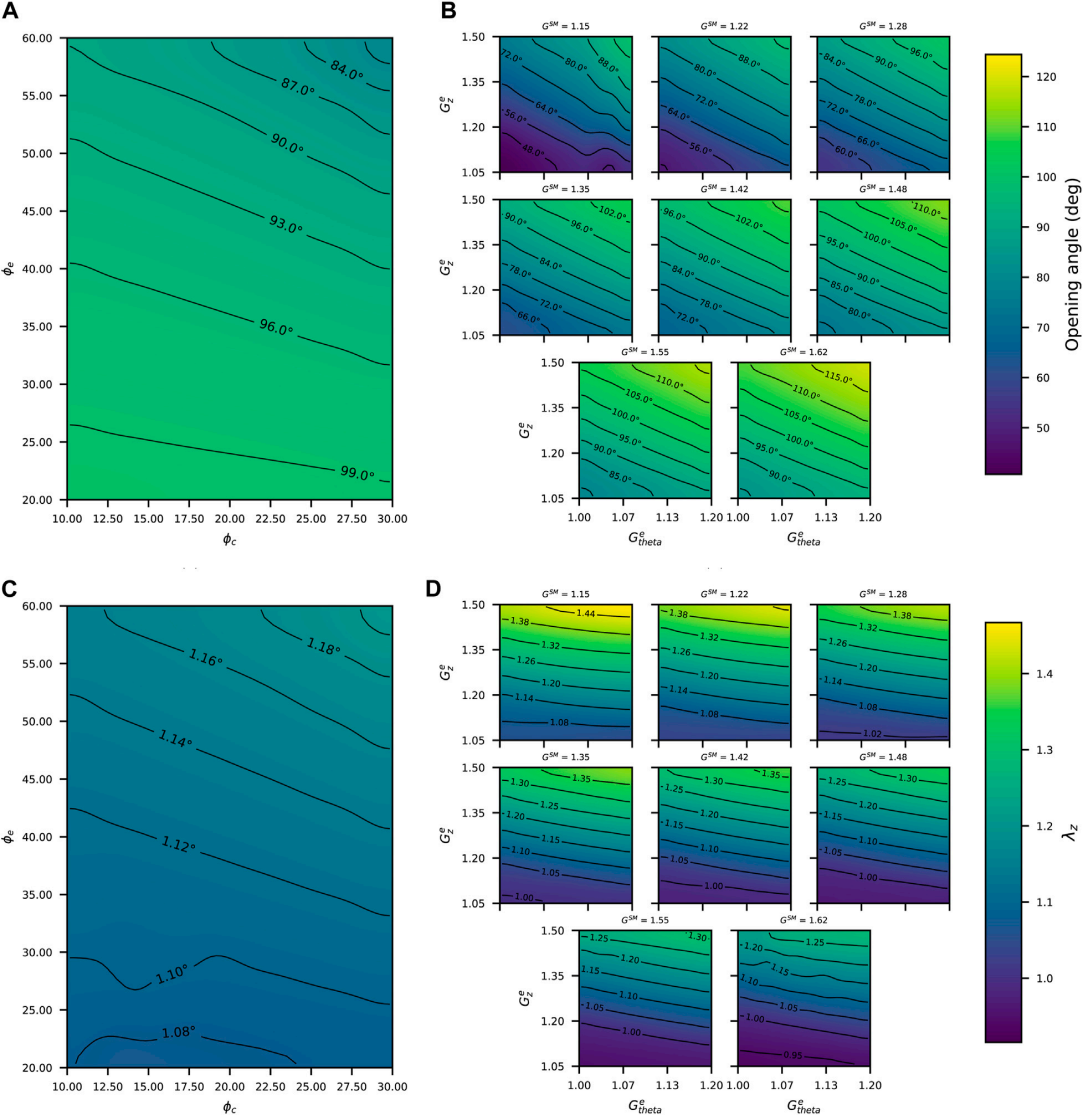
Opening angle in function of: **(A)** mass fractions; **(B)** deposition stretches. Longitudinal stretches *λ*_z_ in function of **(C)** mass fractions; **(D)** deposition stretches.

Figure 7 displays all the results from this study, according to the different cases considered. In particular, Figure 7A shows the opening angle *α* by varying the *ϕ*_e_ and *ϕ*_c_ values. According to the range of the variation of these mass fractions, there is an inverse relationship between them and the opening angle reached. Specifically, the highest values of *ϕ*_e_ and *ϕ*_c_ considered in this study have resulted in an opening angle of ≈80° ; meanwhile, when both mass fractions adopt the minimum values, the opening angle is close to 100°. Figure 7B also shows the opening angle, this time with respect to the variation of $G_{\theta }^{\text{e}}$ , $G_{\text{z}}^{\text{e}}$ and G^m^. For the six graphs displayed (each one with a constant value of G^m^), the same trend can be observed, which is referred to an increment of the opening angle as the remaining deposition stretches ( $G_{\theta }^{\text{e}}$ and $G_{\text{z}}^{\text{e}}$ ) increase. The global minimum opening angle (≈40°) is given by the lowest combination of deposition stretches, meanwhile at the opposite condition, the highest opening angle (close to 120°) is given when the deposition stretch values go up to the maximum level within the range set in this study. Figure 7C shows the longitudinal stretch (*λ*_z_) according to different combinations of elastin and collagen mass fractions. From these results, an inverse pattern in respect to those shown in Figure 7A can be observed, since higher values of the response variable are visualized in the face of an increment of *ϕ*_e_ and *ϕ*_c_. Moreover, the range of *λ*_z_ variation is bounded between close values ( ≈1.08 to ≈1.20 ). Finally, Figure 7D indicates a colormap of *λ*_z_ values in function of the same deposition stretch values described in Figure 7B. A similar trend concerning the former figure can be observed in the face of an increase of longitudinal stretch to high elastin deposition stretch levels ( $G_{\theta }^{\text{e}}$ and $G_{\text{z}}^{\text{e}}$ ). For a G^m^ variation, it can be observed that when its value is reached, the range of *λ*_z_ is getting lower. To express this trend numerically, *λ*_z_ varies between ≈1.08 and ≈1.44 to G^m^ = 1.15; whereas to the highest smooth muscle deposition stretch (G^m^ = 1.68), the longitudinal stretch is ranged between ≈0.95 and ≈1.25 .

## 4 Discussion

A relevant aspect related to the CMM, particularly referred to the consideration of the *in-vivo* state as the referential configuration, is that it allows its applicability in a clinical context, where non-invasive techniques, such as Computed Tomography (CT) and Magnetic Resonance Imaging (MRI), are capable of measuring the *in-vivo* arterial geometry (Di Cesare et al., 2016). In these circumstances, its implementation in the FE context arises as an essential tool, allowing to predict the mechanical stress field on the arterial wall, under different configurations (for instance *in-vivo*, *ex-vivo* and zero pressure) (Mousavi and Avril, 2017). Regarding the features of CMM, one of the main problems is the lack of information, due to experimental limitations, about the mechanical properties and residual deformation at a constituent level. As proposed in this work, numerical procedures are a feasible way to approximate these unknown parameters based on the available experimental information. Consequently, we have applied it to a patient-specific case, and consistency between the obtained parameters with experimental information has been successfully demonstrated.

Focusing the analysis on the specific aspects related to this study, the results displayed in Figure 5 show a fast convergence of both material parameters (mp) and deposition stretches (ds), where after the sixth iteration, the percentage error in all cases is less than the tolerance defined. Concerning the same analysis performed by Maes et al., 2019 (Maes et al., 2019), the convergence to the set of their material parameters is reached after the seventh iteration, with the consideration of five material parameters (in this study, there were six), and known deposition stretch values.

The parameter fitting procedure described in Section 2.3.3 involves a number of challenges to be addressed, aiming to overcome difficulties in the iterative process and reach consistent results, such as is obtained in this study (see Figure 5). A critical issue to be discussed is referred to the suitable choice of parameters, mainly because of the localized optimization nature of the gradient-based optimization algorithms. The starting parameters have been chosen based on those reported in the existing literature to avoid possible non-realistic solutions. Even when the experimental measurement of the ring-opening angle (*α*) does not form part of the optimization process itself, it has been proved that the material parameters along with the deposition stretches naturally affect this residual strain, serving as a consistency verification measurement of the stress-release process experienced in this test. A remark regarding this measurement is the consistency of the curvature across the opened tissue, suggesting possible bias regarding the idealized geometry considered in this work. Nevertheless, considering that the obtained error percentage between the experimental and numerical opening angle for the final iteration is under 5%, we consider this as a successful verification of the fitted parameters.

A physical interpretation of the determined deposition stretch parameters needs to be analyzed. Relating to elastin (e) fibers, the circumferential and longitudinal components of the deposition stretch tensor determine the values reached by the radial direction, given by the incompressibility constraint considered in this formulation. As such, the higher the values of $G_{\theta }^{\text{e}}$ and $G_{\text{z}}^{\text{e}}$ , the lower the radial component $G_{\text{r}}^{\text{e}}$ $\left(1/\sqrt{G_{\theta }^{\text{e}}G_{\text{z}}^{\text{e}}}\right)$ . This last aspect is physically remarkable, since a value of $G_{\text{r}}^{\text{e}}$ well below the unit triggers an excessive thickening of the arterial wall between the *in-vivo* and *ex-vivo* configurations. For this reason, it is debatable that certain authors report excessively high values of $G_{\theta }^{\text{e}}$ and $G_{\text{z}}^{\text{e}}$ (Bellini et al., 2014; Ahmadzadeh et al., 2019), since there is no evidence of such noticeable changes in thickness. According to the experimental results, the thickness ratio between the *in-vivo* and *ex-vivo* states is 0.73, and to maintain a geometric consistency between these configurations, the deposition stretch values of the elastin matrix are strongly bounded. Another relevant aspect to be considered is referred to the deposition stretch values in those constituents that are modeled as fibers (collagen and smooth muscle). In particular, for the smooth muscle (m), due to the consideration of circumferential arrangement with respect to the arterial duct, the established deposition stretch value G^m^ plays a predominant role on diameter variation between different configurations. From the numerical simulations of Stage 2 (Figure 3B), the muscle deposition stretch significantly contributes to achieving the equilibrium state between the physiological loads and the geometry-preserving constraint. Concerning the collagen fibers (c), as they are arranged diagonally in the longitudinal-circumferential plane, the deposition stretch G^c^ value is not directly related to the other directions. Despite establishing relationships between each constituent separately and analyzing their influence, the interaction of the three load-bearing components (e, c and m) will determine the equilibrium state that satisfies all the mechanical and geometrical behaviors experimentally measured.

Given the scarce information of the parameters that have been established in this work and the results obtained in this context, the deposition stretch value of the smooth muscle G^m^ is outstanding, with reference to those typically reported in the literature. Most of the works in this topic take this value in the range of 1.1–1.2 (Karšaj et al., 2010; Bellini et al., 2014; Laubrie et al., 2022), noting that the half-life of this component within the arterial wall is relatively low, and it is in continuous renewal, so it is not subjected to a great extent to the effects of growth and remodeling. However, no concrete experimental study determines a reference in the magnitude of this value, so the value determined in this work further motivates the study of the mechanisms by which the deposition stretch is physically established (Humphrey et al., 2014).

The sensitivity analysis (Section 3.2) is performed to get a better overview of the relative influence of the mass fractions and deposition stretches of each constituent on residual strains (opening angle *α* and axial stretch *λ*_z_), which are easily measurable experimentally. Figure 7, being formulated based on the same geometrical and material parameters, allows linking strain measurements with residual stresses in the circumferential and axial directions. An important aspect to emphasize from these results is that both *α* and *λ*_z_ are more sensitive to the variation in the deposition stretch values than to the mass fractions. That point supports the procedure established in Section 2.3 of this work, regarding the consideration of referential values of mass fraction, while the deposition stretch values were those to be determined within the fitting procedure due to the importance of a correct choice of their values. This aspect reinforces the effectiveness of the iterative fitting procedure, by applying it to a patient-specific case.

In addition, some works related to CMM consider the arterial wall as a two-layer material, according to the fact that the media and adventitia have different composition and material properties (Karšaj and Humphrey, 2012; Mousavi et al., 2019). However, in most animal models taken as reference (including those used in this work), it is not possible to separate both layers experimentally to perform individual mechanical tests. In many cases, the parameters considered are fictitious, and the analysis is focused on numerical purposes. In Latorre and Humphrey, 2018 (Latorre and Humphrey, 2018), however, this distinction let account for differences in medial *versus* adventitial fibrosis that resulted in marked aortic maladaptation in hypertension. In this particular case, as the experimental information available refers to the whole tissue, the CMM considers homogenized parameters along the thickness, avoiding the incorporation of different hypotheses about the characteristic behavior by each specific layer.

The consideration about the behavior of fibers under compressive loads, named in literature as tension-compression switch (Latorre et al., 2016; Horgan and Murphy, 2020), has been implemented in this study, according to the seminal works of Holzapfel et al., 2000 and Gasser et al., 2006 (Holzapfel et al., 2000; Gasser et al., 2006). In this regard, different objections have been raised about this hypothesis, mainly due to the lack of experimental evidence to back it up (Horgan and Murphy, 2020). suggested physical contradictions concerning isotropic response predicted by the constitutive models under a compression state, according to the experimental results provided by Holzapfel 2006 (Holzapfel, 2006). However, there is no clear evidence on the underlying mechanism of the fibers under compression. Some studies, related to the CMM, incorporate the distinction of tension and compression material properties on collagen fibers and smooth muscle (Bellini et al., 2014; Mousavi and Avril, 2017). However, there is no clear experimental evidence to support the values used.

The strain energy function derived within the CMM context (Expression 2) represents a phenomenological approach of arterial structure, through the consideration of isotropic and anisotropic behavior in separated terms (the former representing the integrated influence of elastin (e) and ground substances, and the latter those of collagen (c) and smooth muscle (m)). According to the material parameters (mp) determination (Holzapfel, 2006), identified a critical issue, referred to the impossibility of determining a unique set of values from the information provided only by uniaxial tensile tests, due to the fact that strain state of this mechanical test does not represent the physiological biaxial condition. To overcome this problem, it is necessary to obtain additional information about the microstructural arrangement of the arterial wall via histological analysis in order to characterize a physically-consistent set of material parameters as well as to include more experimental data from uniaxial or other experimental tests (Latorre et al., 2016). Recent investigations have focused on determining and evaluating non-destructive techniques for measuring arterial wall constituents, related directly to the determination of collagen fiber orientation and its reorientation in the face of external loads. Several methods have been used by different authors, as for instance, the second harmonic generation (SHG) (Deniset-Besseau et al., 2010; Bancelin et al., 2012; Bancelin et al., 2014; Golaraei et al., 2019; Cavinato et al., 2020), polarized spatial frequency domain imaging (pSFDI) (Jett et al., 2020) and quantitative-polarized light microscopy (Q-PLM) (Greiner et al., 2021), establishing an interesting possibility to switch from a phenomenological approach to a microstructural-consistent one.

In general, the incompressibility of the arterial wall is commonly accepted, and it has been widely taken as a hypothesis in multiple numerical formulations. However, some authors questioned this consideration, reporting volume changes in the physiological pressure range (Yosibash et al., 2014; Yossef et al., 2017). The work of Nolan et al., 2014 (Nolan and McGarry, 2016) performed on an ovine aorta revealed a degree of compressibility, given by a Poisson’s ratio value of 0.44. Therefore, constitutive models of compressible nature could affect the accuracy of stress prediction (Skacel and Bursa, 2019).

## 5 Conclusion

In order to assess the practical applicability of the CM model, through the determination of consistent parameters describing the mechanical response of the arterial wall, a numerical-experimental framework based on the finite element model has been established and applied to a patient-specific case. A fitting algorithm has been implemented to achieve this goal, considering experimental information of the *in-vivo* and *ex-vivo* geometries, along with data from uniaxial tensile tests in two directions. This information is set as a target, and by the application of an inverse finite element problem procedure, the material parameters (mp) and deposition stretch (ds) values are determined. The convergence of the procedure is evident, and the consistency of these parameters is reflected in the good relationship between experimental and numerical data. On the other hand, a parameter sensitivity study has established the preponderance of the deposition stretch values concerning the residual deformation levels. It should be noted that the CM model is formulated to model G&R phenomena, so the parameter determination through the procedure established in this work is contextualized as a starting point for the incorporation of these effects in the different numerical models to be developed.

## Data Availability

The raw data supporting the conclusion of this article will be made available by the authors, without undue reservation.
